# Identification of pivotal genes and pathways in Chorea-acanthocytosis using comprehensive bioinformatic analysis

**DOI:** 10.1371/journal.pone.0309594

**Published:** 2024-09-18

**Authors:** Ravinder Sharma, Kiran Yadav, Leeza Monga, Vikas Gupta, Vikas Yadav

**Affiliations:** 1 Faculty of Pharmaceutical Sciences, The ICFAI University, Baddi, Himachal Pradesh, India; 2 Department of Clinical Research, National Institute of Pharmaceutical Education and Research, S.A.S. Nagar, Mohali, Punjab, India; 3 University Centre of Excellence in Research, Baba Farid University of Health Sciences, Faridkot, Punjab, India; 4 Department of Translational Medicine, Clinical Research Centre, Skåne University Hospital, Lund University, Malmö, Sweden; Saveetha University - Poonamallee Campus: SIMATS Deemed University, INDIA

## Abstract

Chorea-acanthocytosis (ChAc), an autosomal recessive disorder, is associated with cognitive and behavioral abnormalities. Previous studies were focused around exploring the functional annotation of VPS13A gene in ChAc, whereas the genetic labyrinth underlying this disease and plausible drug targets were underexplored. In the present study, we have identified the pivotal genes and molecular pathways implicated in ChAc using comprehensive bioinformatics analysis. In our analysis we found 27 distinct genes in Homo sapiens linked to ChAc, out of which 15 were selected as candidate genes for enrichment analysis based on their Gene Ontology (GO) annotations and involvement in relevant molecular pathways. By constructing a Protein-Protein Interaction (PPI) network consisting of 26 nodes and 62 edges, we identified two gene modules. Subsequently, using the MCODE algorithm, we identified 6 hub genes—ATN1, JPH3, TBP, VPS13A, DMD, and HTT—as core candidates. These hub genes are primarily associated with processes such as neuron development and differentiation, the CAMKK-AMPK signaling cascade, ion transmembrane transport systems, and protein localization. Furthermore, using drug gene databank we identified 23 FDA-approved drugs that possess the propensity to target 3 out of the 6 identified hub genes. We believe that our findings could open promising avenues for potential therapeutic interventions in ChAc.

## Introduction

The neurological condition chorea-acanthocytosis (ChAc) is characterized by irregular red blood cell (RBC) morphology and repetitive motions of different body segments. Since there have only been a few 1000 cases reported worldwide, researchers and clinicians typically classify ChAc as a rare genetic disease. Previous studies have suggested that the genetic defect causing ChAc may be due to autosomal-recessive mutations in the Vacuolar Protein Sorting 13 homolog A (VPS13A) gene [1–3]. Even though this disease causes considerable morbidity, markedly reduces lifespan, and severely affects one’s ability to make decisions, it is still incurable [4]. Patients with ChAc frequently show movement problems resembling those of chorea, Parkinsonism, and/or dystonia. The clinical phenomenology of ChAc is incredibly diverse [5–7]. Additionally, symptoms such as dysarthria and dysphagia, peripheral neuropathy, epilepsy, or cognitive impairment may be present. Some more specific signs of ChAc include tongue and lip biting, self-mutilating behavior, feeding dystonia, or head drops [5–7]. Conclusively, ChAc belongs to the group of Huntington’s disease phenocopies [8]. ChAc is primarily diagnosed by measuring creatine kinase (CK) levels, serum neurofilament (sNfL) levels, and RBC acanthocytosis [5, 7, 9, 10]. In correlation with the clinical manifestations, the main histopathological characteristics include the loss of striatal medium spiny neurons and distinct cortical neurodegeneration [11, 12]. Based on all this information, there is a clear and pressing necessity to identify reliable biomarkers and therapeutic targets for the clinical management of patients dealing with ChAc. In recent years, integrative methods combining multiple data sources have become popular among researchers for identifying genes in complex or rare disorders. Network modeling of gene-gene and/or protein-protein interaction also offers new insights into understanding and identifying disease-related factors. In the present study, using biological databases and the existing published literature we tried to explore the genetic labyrinth of ChAc.

Recent studies have shown that inhibition of Lyn kinase with Dasatinib or upregulation of store operated Ca^2+^ entry (SOCE) with Lithium may serve as a plausible disease modifying therapeutic options. Interestingly, both approaches highlight the concept of “Drug Repositioning”, a strategy previously shown to be beneficial for numerous orphan or rare diseases. “Drug Repositioning” refers to finding a new implication for an existing medication to treat a condition other than its intended implication [13–15]. With the aim of identifying a probable drug candidate for ChAc we performed text mining of biomedical literature and amalgamated it with the Drug-gene interaction database. Our analysis revealed a few probable drug candidates that could become a game changer in the management of ChAc patients, but it is subject to further clinical validations in future.

## Methods

### Identification of ChAc associated key genes using text mining analysis

**Fig 1:**
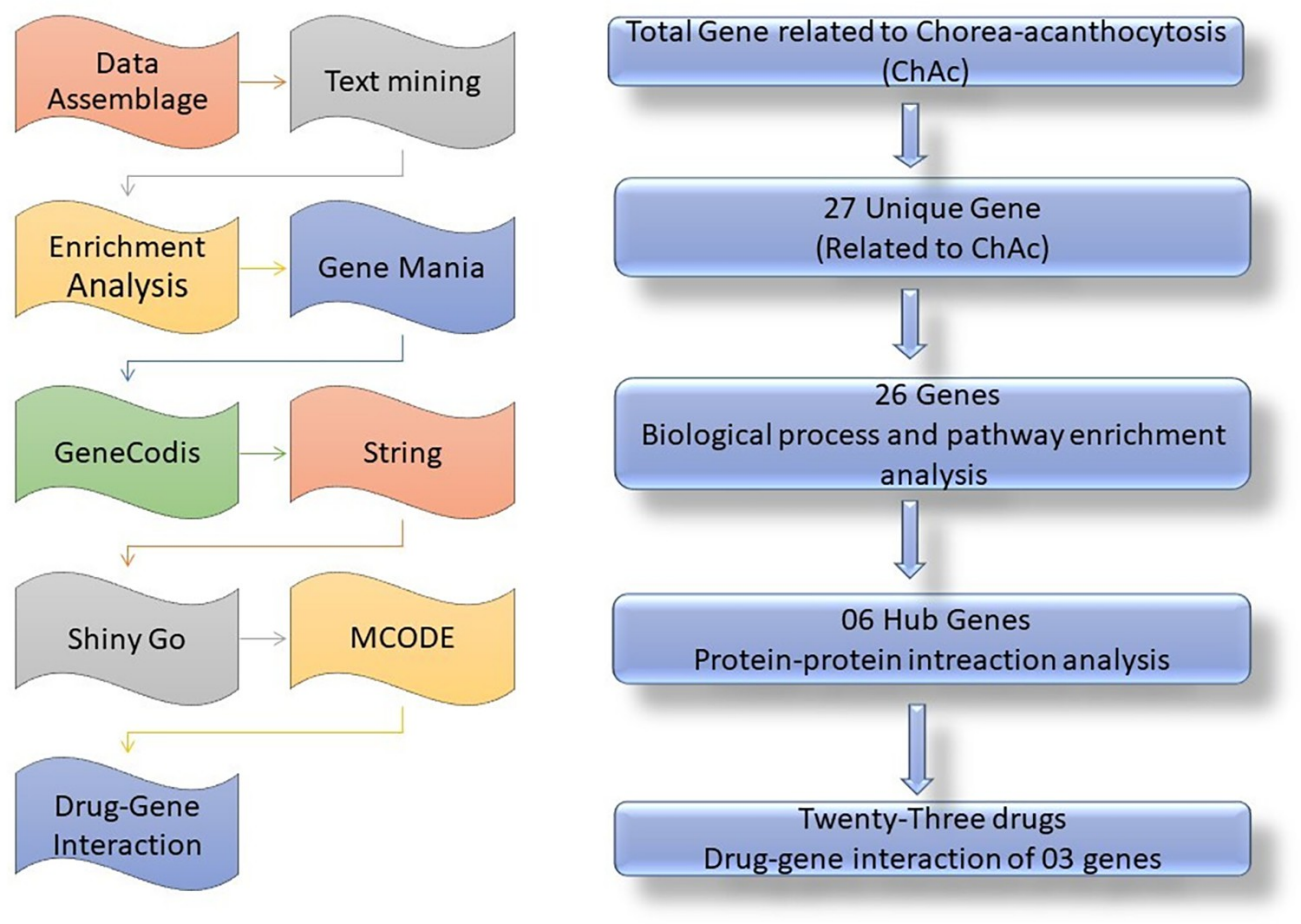
A synopsis of the research design and flowchart.

Pubmed2ensembl tool (http://pubmed2ensembl.ls.manchester.ac.uk) was used for text mining analysis to assess the relationship between genes and the literature for data extraction, to find genes associated with ChAc. Pubmed2ensembl tool is a publicly available database that linked 150,000 Ensembl genes from 50 species to approximately 2,000,000 articles in PubMed journals [16, 17]. We employed the search phrase "Chorea Acanthocytosis" and "Choreoacanthocytosis" to extract a list of important genes from 100,000 pertinent document IDs. To prevent genes linked to other neurological illnesses from overlapping, the search terms were restricted. "Homo sapiens" was selected as the species dataset, and "MEDLINE: PubMed ID" was used to constrain the query result. After extracting the unduplicated genes, the intersection of gene hits from the two sets was used to identify the TMGs. The approach flowchart and research design overview are shown in Fig 1. The data from all the databases were accessed between January to March 2024.

### Pathway enrichment and biological process analysis

Using the web-based platform GeneCodis, the text mining-derived TMGs were examined for biological process annotations [18]. The genes implicated in the enrichment process were subsequently examined for functional relationships by selecting genes with significantly enriched biological processes related to ChAc, using the TMGs as input [19]. Using the Cytoscape plugin version 3.9.1, GeneMania (version 3.5.2) was utilized to create a gene-gene functional interaction network from the TMGs. The criteria for determining significant enrichment and functional relationships were based on adjusted p-values (FDR < 0.05) for GO and pathway enrichment analyses. These criteria ensure that the observed enrichments are statistically significant and not due to random chance. We have clarified these criteria in the Methods section.

### Creation of Protein-Protein Interaction (PPI) network and module assessment

The PPI network comprising 26 enriched genes was built using STRING (version 11.5) and was based on GO. STRING is an online database that includes more than 3.1 billion interactions and around 24.6 million proteins from 5,090 different species [https://string-db.org/cgi/input.pl]. In network theory, various metrics are used to analyse the properties and significance of nodes within a network which are as follows: Degree centrality (k), which measures the number of connections a node has, indicating its immediate importance in the network; Betweenness centrality (BC), which measures the number of times a node acts as a bridge along the shortest path between two other nodes, indicating its role in facilitating communication within the network; Closeness centrality (CC), which measures how close a node is to all other nodes in the network, indicating its overall reach and influence; Eigenvector centrality (EC), which measures a node’s influence based on the importance of its neighbours, indicating nodes that are connected to other highly influential nodes; Eccentricity, which measures the maximum distance from a node to any other node, indicating its position relative to the network periphery. Understanding these metrics helps in analyzing the structure and dynamics of networks, identifying key nodes, and assessing the robustness and vulnerability of the network. Nodes with a high BC, known as bottlenecks, prefer to represent important genes because they can be compared to heavily trafficked intersections on major highways or bridges, hub proteins, or nodes with a high degree, are crucial proteins in the PPI network because they may correlate to genes that cause disease. The minimal requirement was stated as a confidence score of 0.900. This high threshold ensures that the interactions included in the PPI network are highly reliable, as they are supported by strong experimental and computational evidence. This choice enhances the robustness and accuracy of our network analysis. The Cytoscape program, which graphically displays the integration of gene expression, biological network, and genotype, was then used to analyze the molecular interaction network and identify hub genes [20, 21]. The sub-network of these important proteins was regarded as the backbone of the study, which merited further investigation in the signaling pathways involved in eye development. The hub nodes were categorized by a high score based on the network’s scale-free property and were used for centrality analysis by examining the network topology [22]. We used MCODE (Molecular Complex Detection, version 2.0.0), a built in Cytoscape plugin with default parameters to identify densely connected modules in the PPI network. Hub genes were identified based on their high MCODE scores, which indicate highly interconnected nodes. Additionally, nodes with a degree centrality above the 90th percentile were also considered hub genes [23]. "Degree cut off = 2," "Node score cut off = 0.2," "k-core = 2," and "max depth = 100" were the cut off settings [24].

### Drug-gene interactions

An online tool called the Drug-Gene Interaction Database (DGIdb) (www.dgidb.org) compiles information from multiple sources to show gene drug ability and drug-gene interactions [25]. Using DGIdb (Version 4.2.0), we examined drug-gene interactions utilized in important module genes as possible targets for currently available medications or chemicals. The chemical structures of the recognized medications were obtained using the PubChem database (https://pubchem.ncbi.nlm.nih.gov). It has 90 million bioactivity outcomes connected to thousands of macromolecular targets, and more than 25 million distinct chemical structures.

## Results

### Determination of potential genes

**Fig 2:**
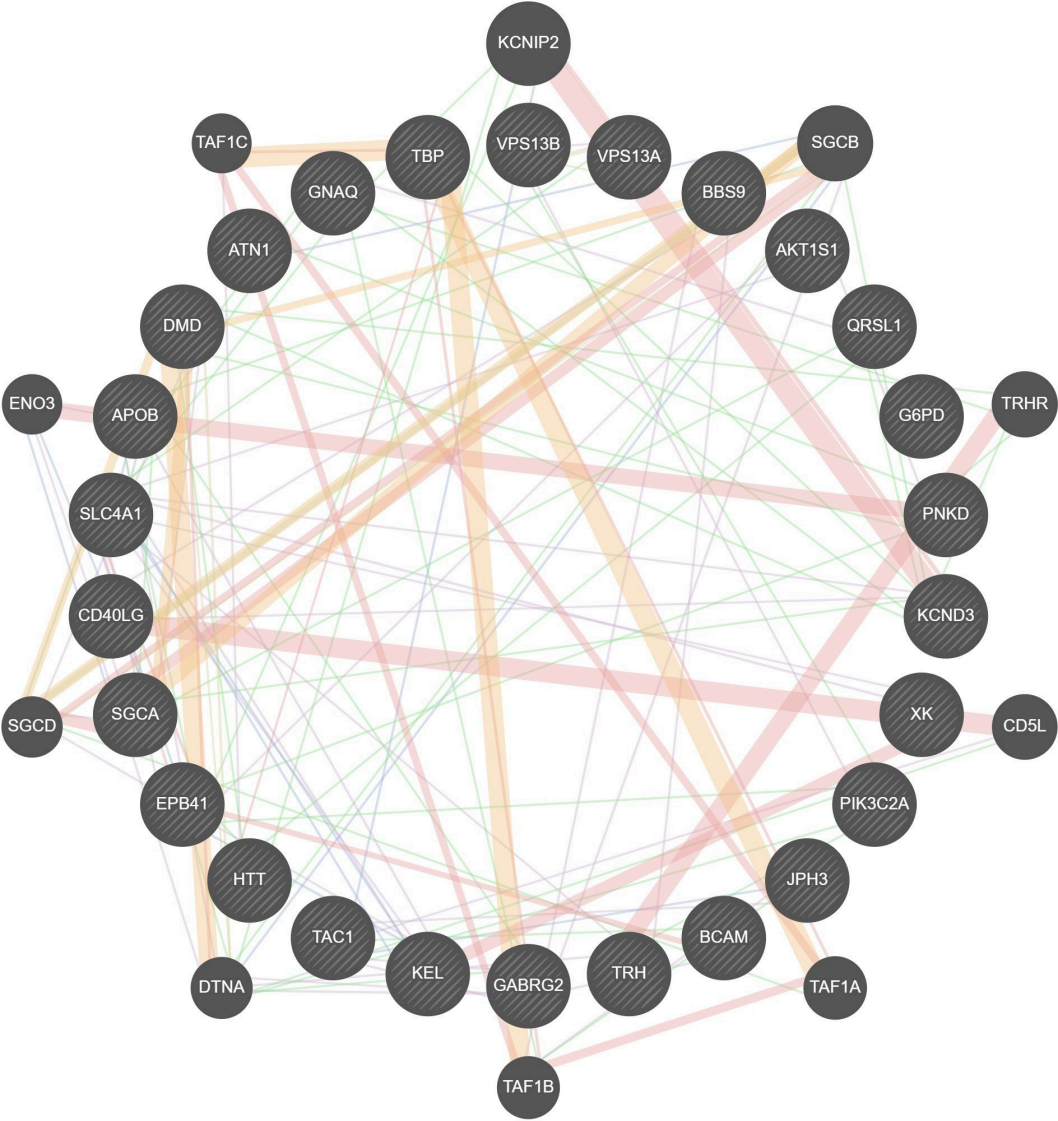
PPI network of all text mining genes (TMG) related to ChAc.

By employing the TMG approach, we acquired 27 unique genes in Homo sapiens associated with ChAc. Fig 2 depicts the network, genetic interactions, co-expression analysis and pathways of the 27 TMGs assessed by GeneMania. From these, 15 genes were selected as candidate genes for enrichment analysis based on their GO and molecular pathways.

### Enrichment analysis of TMG

Through the use of GeneCodis, GO, biological process (BP), and KEGG, the most enriched terminology that was directly related to the pathophysiology of ChAc was found. The examination of GO and BP annotations revealed 15 genes that were considerably enriched. The 10 most enriched functions were “Cellular magnesium ion homeostasis” (P = 2.99E-03), “Regulation of axon diameter” (P = 2.99E-03), “Regulation of neuron apoptotic process” (P = 3.18E-02), “Regulation of ryanodine sensitive calcium release channel activity” (P = 3.18E-02), “Skeletal muscle fibre development” (P = 3.18E-02), “Regulation of cell size” (P = 3.18E-02), “Triglyceride mobilization” (P = 4.14E- 02), “Negative regulation of protein glutathionylation” (P = 4.14E-02), “Regulation of cAMP dependent protein kinase activity” (P = 4.14E-02), and “Positive regulation of protein localization to cell cortex” (P = 4.14E-02). Using KEGG enrichment analysis, 15 main pathways involving 10 TMGs were found overall. The five most significantly enriched pathways were “Viral myocarditis” (P = 2.01E- 02), “Spinocerebellar ataxia” (P = 1.33E-01), “Dilated cardiomyopathy” (P = 2.96E-01), “Arrythmogenic right ventricular cardiomyopathy” (P = 2.96E-01), and “hypertrophic cardiomyopathy” (P = 2.96E-01), involving 3, 3, 2, 2, and 2 text mining genes, respectively. Table 1 shows 15 enriched GO terms, and Table 2 shows the KEGG analysis of 10 enriched molecular pathways of the TMGs.

**Table 1:** Top 15 biological process concepts from GO that are enriched and linked to the TMGs.

Biological Process	Genes in query set	Total genes in the genome	P-value	Genes
cellular magnesium ion homeostasis	2	5	2.99e-03	KEL, XK
regulation of axon diameter	2	5	2.99e-03	KEL, XK
regulation of neuron apoptotic process	2	22	3.18e-02	AKT1S1, G6PD
regulation of ryanodine-sensitive calcium-release channel activity	2	19	3.18e-02	DMD, JPH3
skeletal muscle fiber development	2	26	3.18e-02	KEL, XK
regulation of cell size	2	24	3.18e-02	KEL, XK
triglyceride mobilization	1	4	4.14e-02	APOB
negative regulation of protein glutathionylation	1	2	4.14e-02	G6PD
regulation of cAMP-dependent protein kinase activity	1	4	4.14e-02	HTT
positive regulation of protein localization to cell cortex	1	5	4.14e-02	EPB41
regulation of muscle system process	1	2	4.14e-02	DMD
NADPH regeneration	1	4	4.14e-02	G6PD
regulation of skeletal muscle contraction by regulation of release of sequestered calcium ion	1	3	4.14e-02	DMD
peptide biosynthetic process	1	4	4.14e-02	DMD
sperm mitochondrion organization	1	2	4.14e-02	VPS13A

**Table 2:** Top 10 enriched KEGG pathways assigned to the TMGs.

ChAc-KEGG pathway	Genes in the query set	Total genes in the genome	P-value	Genes
Viral myocarditis	3	59	2.01e-02	CD40LG, DMD, SGCA
Spinocerebellar ataxia	3	143	1.33e-01	GNAQ, KCND3, TBP
Dilated cardiomyopathy	2	95	2.96e-01	DMD, SGCA
Arrhythmogenic right ventricular cardiomyopathy	2	77	2.96e-01	DMD, SGCA
Hypertrophic cardiomyopathy	2	90	2.96e-01	DMD, SGCA
Huntington disease	3	293	3.19e-01	GNAQ, HTT, TBP
Intestinal immune network for IgA production	1	47	3.28e-01	CD40LG
Growth hormone synthesis, secretion and action	1	119	3.28e-01	GNAQ
Basal transcription factors	1	45	3.28e-01	TBP
Cholinergic synapse	1	113	3.28e-01	GNAQ

### PPI network construction, modular analysis, and key genes identification

**Fig 3:**
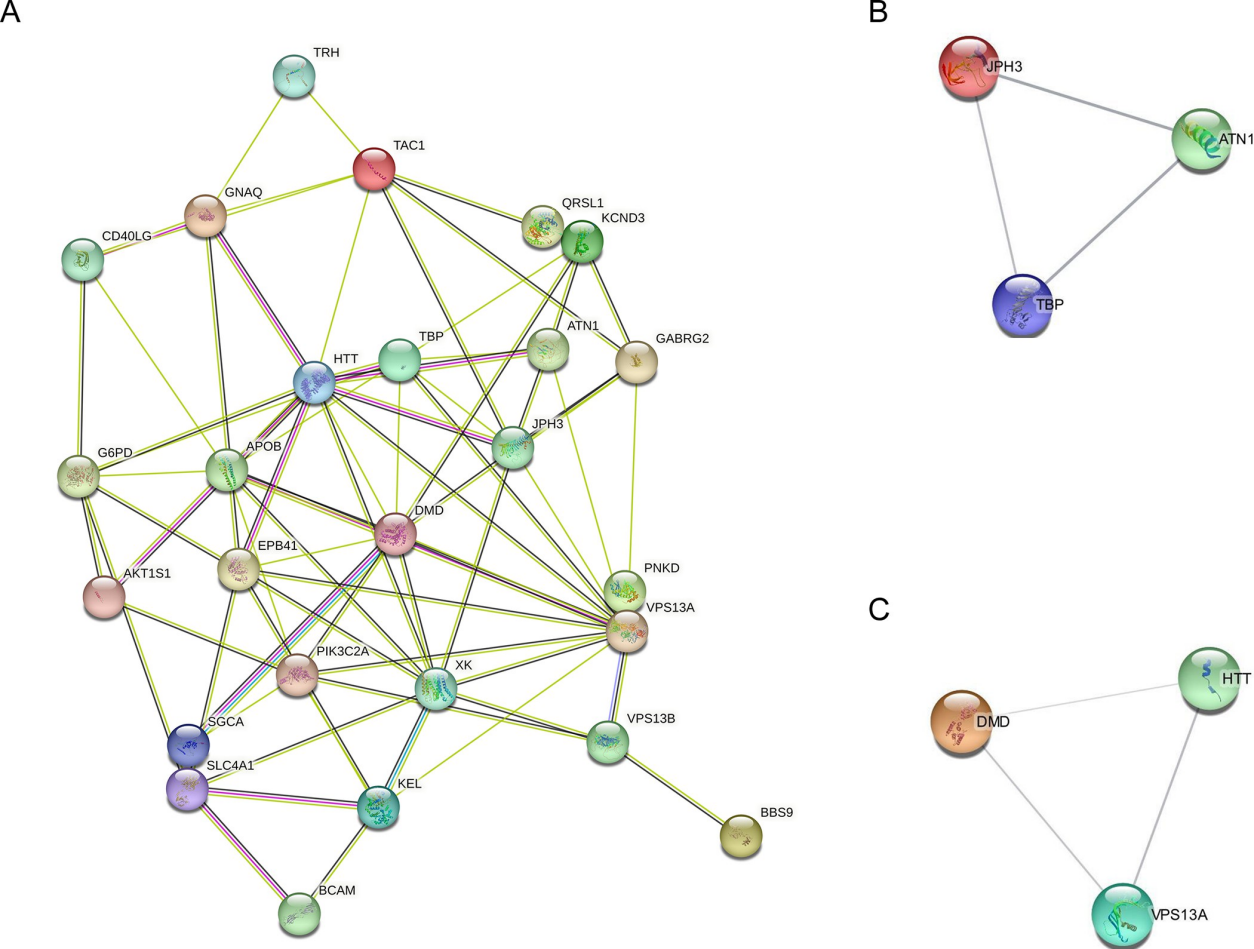
Identification and enrichment analysis of the TMGs. **(A)** PPI network of the 26 target TMGs as visualized by Cytoscape. **(B, C)** Using MCODE, the two modules were retreived from the PPI network, namely, Module 1 and Module 2. Both the modules were having 3 nodes each.

**Fig 4:**
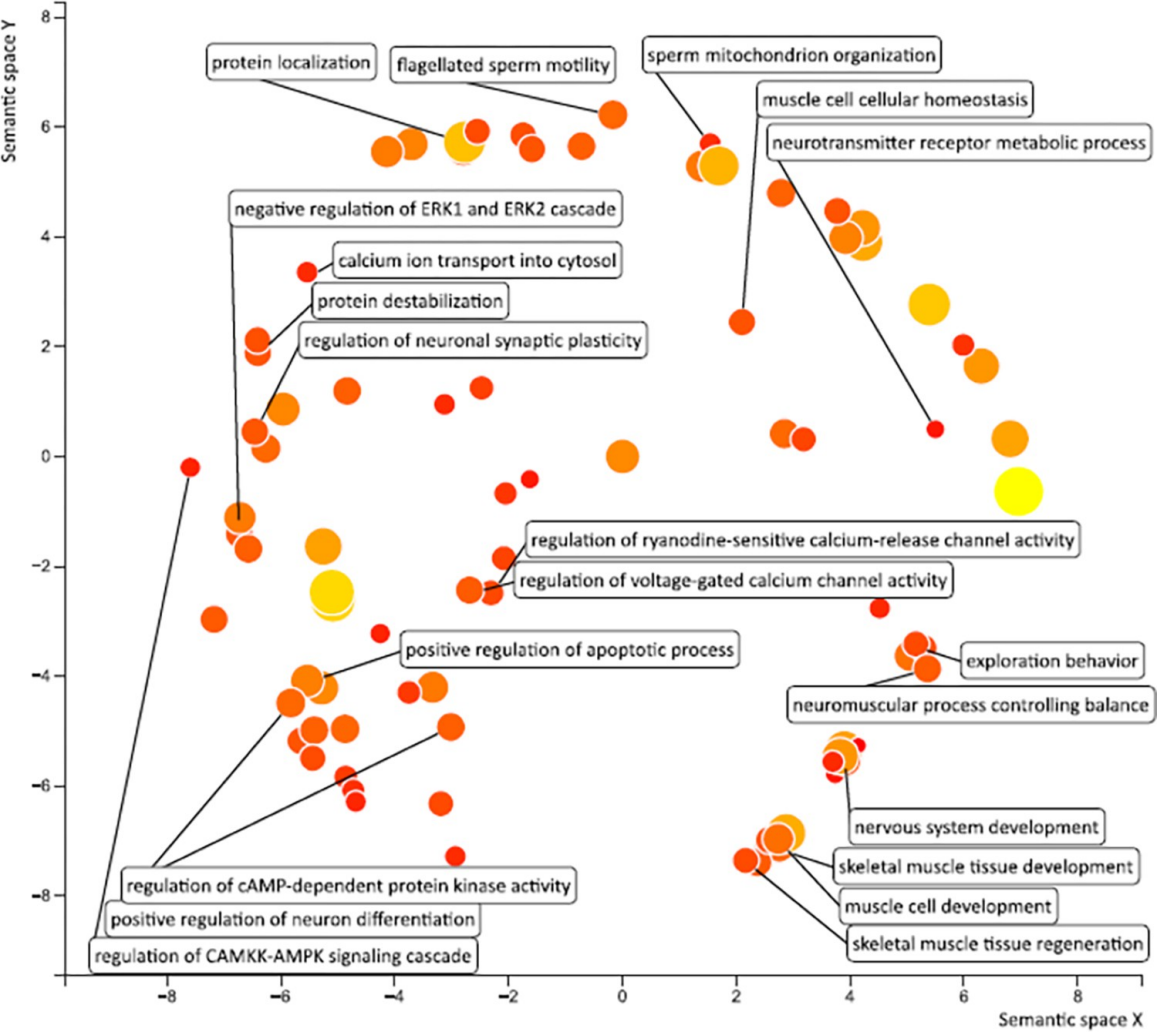
Gene Ontology terms of the 6 hub genes. The enriched GO keywords associated with the nervous system and protein localization are depicted in the image. We used DAVID and the REVIGO web server to perform route and functional enrichment analysis.

**Fig 5:**
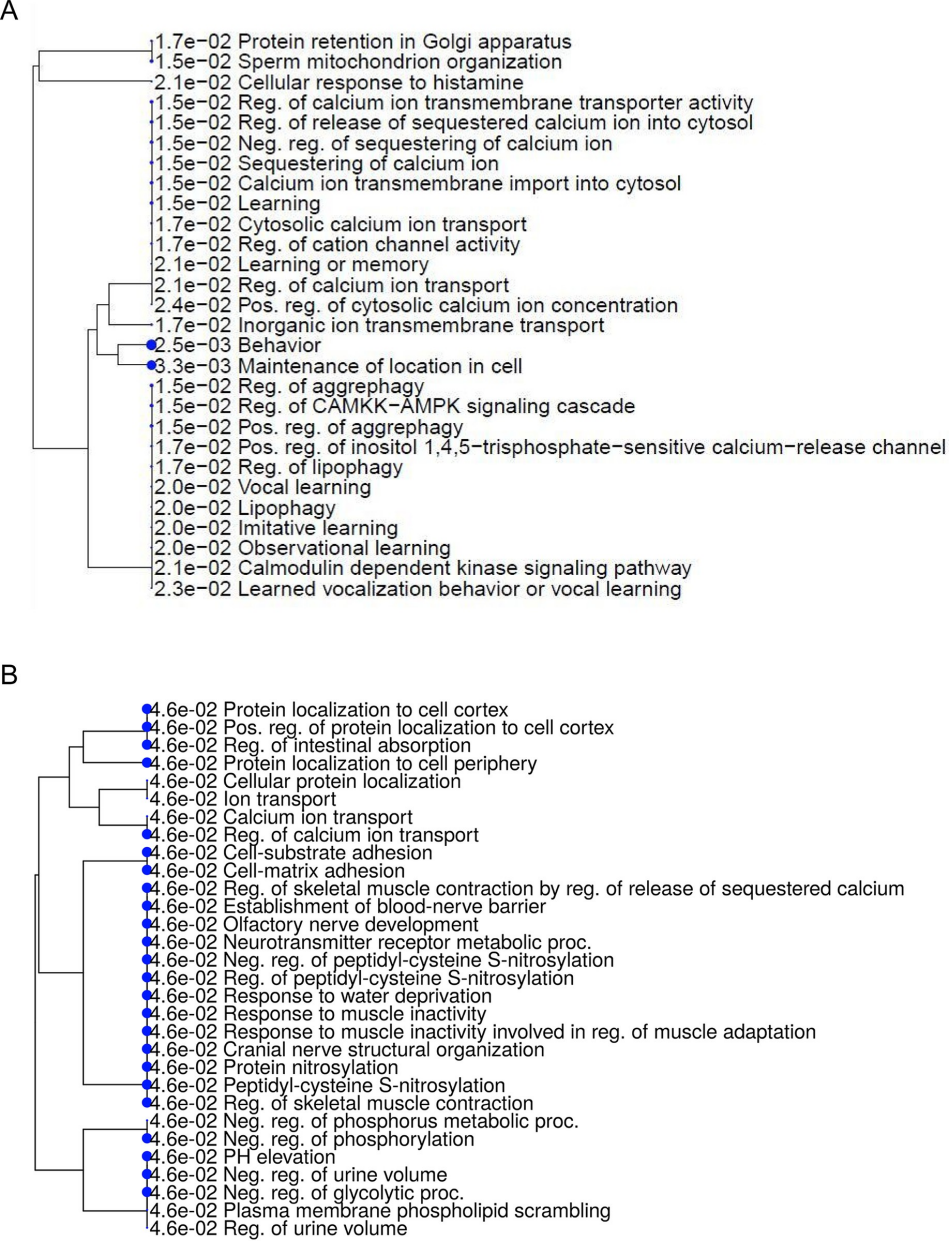
Gene Ontology (GO) terms in the two modules. **(A)** Module-1’s significantly enriched GO keywords. **(B)** Module-2’s significantly richer GO keywords. A tree depiction made with the ShinyGo web server showed a strong correlation (P > 0.005) between the functional and route enrichment studies in ChAc.

As shown in Fig 3, for each of the 26 target genes, a PPI network with a low confidence score of less than 0.200 was built using STRING. The network consisted of 68 edges and 26 nodes (Fig 3). Six hub node genes were found among 26 nodes using a cluster analysis of filtering nodes (Table 3). The hub genes that were found were VPS13A, JPH3, DMD, HTT, ATN1, and TBP. Based on GO similarity, the REVIGO analysis of the hub genes identified eight clusters that were mostly associated with the development of skeletal muscle tissue, the neuromuscular process that regulates balance, protein processing, calcium ion transport regulation, and nervous system development (Fig 4). Two modules were produced from the modular analysis done with MCODE. Because module 1 (ATN1, TBP, and JPH3) and module 2 (DMD, HTT, and VPS13A) each contained three genes, the PPI network is dependent on a total of three genes. Based on the pathway enrichment study conducted with KEGG and the ShinyGo platform, the genes included in module 1 were linked to memory, calcium ion transport regulation, behavior, and cell location maintenance. The regulation of skeletal muscle contraction, cell adhesion, and protein localization were found to be substantially correlated with the module 2 genes (Fig 5). All things considered, the enrichment analysis showed that these genes were significantly enriched in ion transport, cell morphogenesis, and developmental process regulation—all of which are critical for neuronal differentiation in ChAc.

**Table 3:** Hub node genes in the PPI network identified with a filtering node degree = 2.

Genes	Degree	MCODE Clusters	MCODE Node Status	MCODE Score
TBP	2	Clustered	Module 1	3.4285714
JPH3	2	Clustered	Module 1	3.4285714
ATN1	2	Clustered	Module 1	4
VPS13A	2	Clustered	Module 2	2.8888888
DMD	2	Seed	Module 2	3.1428571
HTT	2	Clustered	Module 2	2.8888888

### Drug-gene interaction analysis of core genes

Six hub genes were chosen as possible therapeutic targets in the drug-gene interaction investigation. In all, three out of the six could be gene targets, and drug-gene interactions are anticipated for twenty-three FDA-approved medications (Table 4). The exceptions were JPH3, ATN1, and VPA13A. Eteplirsen and Ataluren are drugs designed to address the underlying genetic mutations in DMD gene, which works by altering exon splicing and promoting dystrophin expression. Eteplirsen is an antisense oligonucleotide that induces exon skipping during pre-mRNA splicing of the DMD gene, allowing the production of a truncated but functional dystrophin protein. Whereas Ataluren promotes read-through of premature stop codons during translation, potentially restoring the production of full-length dystrophin.

**Table 4:** Information on the 23 Food and Drug Administration (FDA)-approved drugs that likely target three of the six hub genes.

Drug	Chemical Formula	Gene	Interaction	Interaction Score	Drug Class	Approved	Reference (PubMed Id)
Etoposide phosphate	C_29_H_33_O_16_P	TBP	n/a	17.67	Anticancer agent (plant Alkaloid)	Yes	1646402
Fluvoxamine Maleate	C364-H569-N177-O122-P30	HTT	Inhibitor	0.31	Antidepresant (SSRIs)	Yes	n/a
Desvenlafaxine Succinate	C_15_H_9_FN_2_O_3_	HTT	Inhibitor	0.16	Antidepresant (SNRIs)	Yes	n/a
Vilazodone Hydrochloride	C_15_H_21_F_3_N_2_O_2_	HTT	Inhibitor	0.16	Antidepresant (SSRIs)	Yes	n/a
Clomipramine Hydrochloride	C_16_H_25_NO_2_	HTT	Inhibitor	0.1	Tricyclic Antidepressant	Yes	n/a
Ketorolac Tromethamine	C_26_H_27_N_5_O_2_	HTT	n/a	0.1	NSAIDs	Yes	n/a
Duloxetine Hydrochloride	C_19_H_23_ClN_2_	HTT	Inhibitor	0.08	Antidepressant (SSNRIs)	Yes	n/a
Amitriptyline Hydrochloride	C_15_H_13_NO_3_	HTT	Inhibitor	0.06	Tricyclic Antidepressant	Yes	n/a
Trazodone Hydrochloride	C_18_H_20_ClNOS	HTT	Inhibitor	0.06	Serotonin Modulator	Yes	n/a
Fluoxetine Hydrochloride	C_20_H_24_ClN	HTT	Inhibitor	0.05	Antidepressant (SSRIs)	Yes	n/a
Imipramine Hydrochloride	C_19_H_23_Cl_2_N_5_O	HTT	Inhibitor	0.05	Tricyclic Antidepressant	Yes	n/a
Paroxetine Hydrochloride	C_17_H_19_ClF_3_NO	HTT	Inhibitor	0.03	Antidepressant (SSRIs)	Yes	n/a
Terazosin	C_19_H_25_ClN_2_	HTT	n/a	0.03	Alpha Blocker	Yes	n/a
Chloroxine	C_19_H_21_ClFNO_3_	HTT	n/a	0.03	Antibacterial Agent	Yes	n/a
Leflunomide	C_19_H_25_N_5_O_4_	HTT	n/a	0.03	DMARDs	Yes	n/a
Amoxapine	C_9_H_5_Cl_2_NO	HTT	Inhibitor	0.02	Antidepressant	Yes	n/a
Promethazine	C_12_H_9_F_3_N_2_O_2_	HTT	n/a	0.02	Antihistamine	Yes	n/a
Fluspirilene	C_17_H_16_ClN_3_O	HTT	n/a	0.02	Antipsychotic	Yes	n/a
Methylphenidate	C_17_H_20_N_2_S	HTT	n/a	0.01	CNS Stimulant	Yes	29382897
Mitoxantrone	C_29_H_31_F_2_N_3_O	HTT	n/a	0.01	Antineoplastic Agent	Yes	n/a
Vandetanib	C_14_H_19_NO_2_	HTT	n/a	0.01	Kinase Inhibitor	Yes	n/a
Eteplirsen	C_22_H_28_N_4_O_6_	DMD	n/a	41.22	Antisense Oligonucleotide	Yes	n/a
Ataluren	C_22_H_24_BrFN_4_O_2_	DMD	n/a	6.87	Antisense Oligonucleotide	Yes	n/a

## Discussion

**Fig 6:**
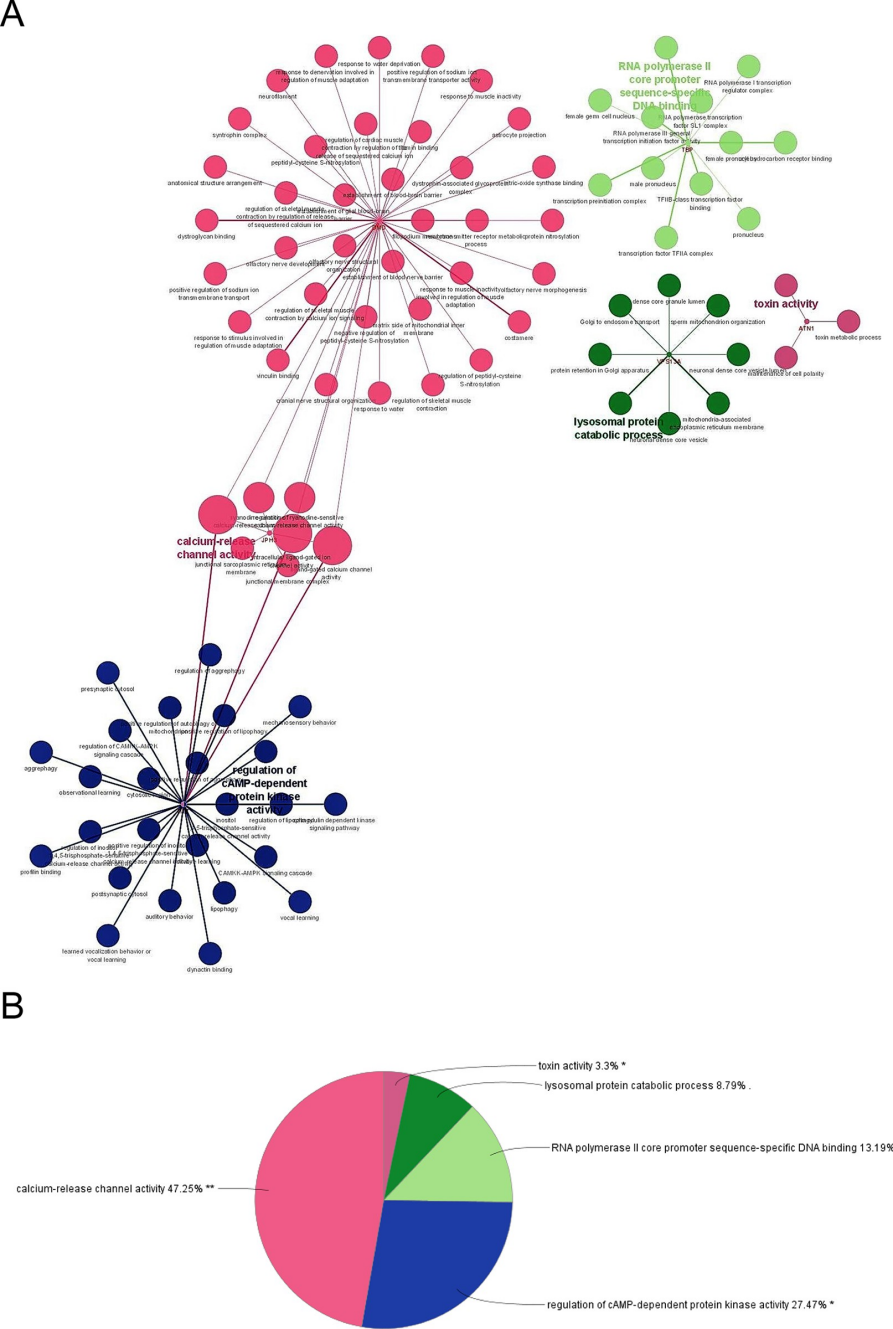
Functional examination of the 6 key significant genes found in modules 1 and 2. (A) ClueGO was used to visualize the main gene’s pathways and functions. (B) The distribution of the core gene’s pathways and functions.

To the best of our knowledge there are currently no known medication that can alter the course of ChAc, a devastating neurological multi-system disease. ChAc is an orphan disease associated with small patient sample group, which limits clinicians’ ability to explore the mechanisms underlying a group of phenotypes. Utilizing traditional variant detection techniques like Next Generation Sequencing or else, which involve evaluating and analyzing molecular pathways and genetic variant analysis, can be costly, time-consuming, and lead to complex data analysis for variations that are not yet identified. Since text mining can uncover previously undiscovered connections between genes and disease pathologies, it is a useful technique for hypothesis generation. Combining text mining with biological knowledge and a bioinformatics methodology offers fresh perspectives on the possibility of repurposing currently available medications. According to our investigation, there are similarities between the genes associated with ChAc and several other signaling pathways, which could contribute towards identifying a wider variety of prognostic biomarkers and therapeutic targets. We and several others have recently reviewed how genomics-based knowledge was instrumental in developing vaccines, diagnostic tools, and personalized treatment plan for recently concluded COVID-19 pandemic [26–28]. It can be presumed that the discovery of biomarkers through genomic research can aid in early detection and monitoring of orphan diseases such as ChAc. Technological advancements such as high-throughput sequencing and improved bioinformatics tools, driven by the pandemic, can be repurposed for studying orphan diseases. Moreover, the knowledge about regulatory processes and ethical frameworks developed for COVID-19 can serve as models for fast-tracking approvals and ensuring responsible research practices for orphan diseases such as ChAc. Our present study identifies 26 genes potentially involved in the pathogenesis of ChAc, with primary associations found in the enriched GO and BP terms related to the modulation of ryanodine-sensitive calcium-release channel activity, cellular magnesium ion homeostasis, skeletal muscle development, regulation of cAMP-dependent protein kinase, and the peptide biosynthesis process. Six hub genes found using the PPI network and enrichment analysis were: VPS13A, HTT, JPH3, ATN1, DMD, and TBP. These genes impact the CAMKK-AMPK signaling cascade, mitochondrial architecture, regulation of ryanodine-sensitive calcium channel function, regulation of skeletal muscle contraction via sequestered calcium ions, and dystroglycan binding (Fig 6). The distributions of functions and pathways among core genes is further shown by the functional analysis and pathways of the important genes in modules 1 and 2, depicted using ClueGO.

As expected, in our study we identified the VPS13A gene which encodes for chorein. In ChAc, the primary issue is a mutation in the VPS13A gene which leads to disruptions in neuronal function and integrity. Unfortunately, its physiological role at the molecular level is still poorly understood. However, the chorein protein is known to contribute towards the maintenance of cytoskeletal structure, ensuring proper cell shape, stability, and transport within cells. Additionally, chorein interacts with lipid droplets and endosomes, implicating it in lipid metabolism and intracellular transport. The main membrane-spanning protein in the bilayer, is connected to the spectrin-actin junctional complex by interactions between chorein, β-adducin, and β-actin. This bridge’s failure causes membrane instability and spontaneous RBC fragmentation, which modifies the RBC’s structure [33]. HTT, another gene identified in our study is not known to be the direct cause for ChAc, but it is known to influence key cellular processes that overlap with those disrupted in this disorder [29]. The huntingtin protein, produced by the HTT gene, supports neuronal health through roles in cytoskeletal dynamics, vesicle trafficking, and mitochondrial function. The brain contains large amounts of HTT, which are repeats of 9–35 trinucleotides that are converted to polyglutamine during protein synthesis. Numerous intracellular processes are disrupted by the overexpression of HTT, which increases the pathogenic trinucleotide repeat beyond 40 [30]. Additionally, this causes the normal protein to lose its function and the mutant huntingtin to toxically gain its function. Moreover, the gradual deterioration seen in ChAc has been linked to excitotoxicity, dopamine toxicity, metabolic impairment, mitochondrial malfunction, oxidative stress, and autophagy [31]. An increasing body of research points to the possibility that a mutation in the HTT gene also affects mitochondrial function. Because mitochondria contain a variety of pro-apoptotic substances that can initiate cell death programs when released into the cytoplasm [32, 33]. The shared involvement of HTT and VPS13A in maintaining cytoskeletal integrity and cellular transport suggests plausible insights into HTT function might help understand the mechanisms underlying ChAc.

The ATN1 gene encodes for Atrophin-1 protein, which, as identified in our study, is localized in both the nucleus and cytoplasm of neurons in the human CNS and is associated with DRPLA (Dentatorubral pallidoluysian atrophy) [34]. Neurodegeneration in DRPLA is primarily caused by the accumulation of mutant ATN1 with an enlarged polyQ tract. This mutation leads to a conformational shift in the protein, causing it to aggregate in neurons. This altered protein accumulation interferes with normal cell functions, leading to neuronal death, which leads to uncontrolled movements and intellectual decline [35]. Similarly, another hub gene, DMD from module-2, encodes for the dystrophin protein, which is primarily present in neurons within specific regions of the CNS and in muscles [36]. Dystrophin is a crucial component of a protein complex that connects the basal lamina to the cytoskeleton. Mutations in various components of the dystrophin protein complex lead to different types of autosomally inherited muscular dystrophies, highlighting the significance of this complex in healthy muscle function. Though its exact function is unknown, it is hypothesized that dystrophin deficiency leads to membrane destabilization and the activation of various pathophysiological processes, many of which converge on changes in the architecture of neuromuscular junctions, intracellular calcium handling, and muscle homeostasis because an increase in intracellular calcium will cause cell necrosis [37]. It is believed that the Tata box binding protein, or TBP, is a universal transcription factor necessary for the start of all three nuclear RNA polymerases [38]. Alteration in this gene influence the transcription of other genes and thus protein synthesis, which ultimately lead to production of defective protein.

In our quest to identify probable drug candidate for ChAc, our drug-gene interaction analysis revealed two potential drug candidates i.e. Eteplirsen and Atalurenfrom. Both drugs have previously shown to promote the expression of DMD by binding to dystrophin mRNA and altering exon splicing. Our this finding is of high interest, as previous studies suggest under-expression of DMD gene to be associated with low levels of dystrophin protein, which leads to myopathy and cell necrosis in ChAc patients [39]. Unlike DMD gene, the overexpression of HTT gene results in increased cell signalling, cell death and aggravation of obsessive-compulsive behaviour [40]. Our results further revealed various class of drugs (Table 4) that could counteract these effects. Since the current work focusses on the evaluating the appropriate pathways and therapeutic options for ChAc through *in silico* analysis, further experimental analysis using animal models is highly recommended to confirm the significance of identified candidate genes and drugs. We believe that our study is of interest related to the field of “Drug Repositioning”. Biopharmaceutical companies throughout the world have already been influenced by the success stories of repositioned compounds, motivating them to adopt similar idea and create their own successful route. Based on present projections, the drug repositioning business is anticipated to have raised roughly $25 billion in 2020 and is expected to reach by $36 billion by 2030 (https://reports.valuates.com/market-reports/QYRE-Auto-16V9136/global-drug-repositioning).

## Conclusion and future perspective

Based on the aforementioned *in silico* investigation using several web servers and software, we have determined that the hub genes ANT1, JPH3, TBP, VPS13A, DMD, and HTT may be implicated in the formation of ChAc. These genes seem to be primarily linked to processes that are involved in the CAMKK-AMPK signaling pathway, control over cAMP-dependent protein kinase activity, binding of dystroglycan, control over the release of sequestered calcium ions during skeletal muscle contraction, and response to muscle activity, which in turn causes neurodegeneration and consequently problems with movement. According to a prior study, under-expression of DMD results in reduced production of dystrophin, which is necessary for muscle function and causes myopathy, whereas overexpression of HTT manifests enhanced neuronal signaling and apoptosis, which causes neurodegeneration. The current work is helpful when there is significant route heterogeneity underlying the clinical phenotype or when there is limited pathological understanding about the disease, like chorea-acanthocytosis is a rare autosomal syndrome. Thus, in addition to analyzing biological pathways specific to individual cases, a combination of therapeutic approaches, including medical intervention and candidate gene identification, may be utilized to suggest possible drug combinations based on gene products annotated to the disease associated with Chorea-acanthocytosis. There are 23 FDA-approved medications that may be used as therapeutic agents to treat and control chorea-acanthocytosis. Our present study focuses on the appropriate path for understanding molecular pathways and therapeutic options for ChAc through *in silico* analysis; additional animal model-based experimental research is strongly advised to validate the importance of the identified candidate genes and medications. We believe that this is the major limitation for our present study.
